# Tracheomalacia and Tracheobronchomalacia in Pediatrics: An Overview of Evaluation, Medical Management, and Surgical Treatment

**DOI:** 10.3389/fped.2019.00512

**Published:** 2019-12-12

**Authors:** Ali Kamran, Russell W. Jennings

**Affiliations:** Department of General Surgery, Harvard Medical School, Boston Children's Hospital, Boston, MA, United States

**Keywords:** tracheomalacia, tracheobronchomalacia, aortopexy, tracheopexy, tracheobronchopexy

## Abstract

Tracheobronchomalacia (TBM) refers to airway collapse due to typically excessive posterior membrane intrusion and often associated with anterior cartilage compression. TBM occurs either in isolation or in association with other congenital or acquired conditions. Patients with TM typically present non-specific respiratory symptoms, ranging from noisy breathing with a typical barking cough to respiratory distress episodes to acute life-threatening events and recurrent and/or prolonged respiratory infections. There are no definitive standardized guidelines for the evaluation, diagnosis, and treatment of TBM; therefore, patients may be initially misdiagnosed and incorrectly treated. Although milder cases of TBM may become asymptomatic as the diameter of the airway enlarges with the child, in cases of severe TBM, more aggressive management is warranted. This article is an overview of the clinical presentation, evaluation, diagnosis, medical management, and surgical treatment options in pediatric tracheomalacia.

## Introduction

Tracheomalacia (TM) refers to an excessive increase in compliance of the trachea, such that the airway is more susceptible to dynamic and/or static collapse; this is distinguishable from intrinsic airway stenosis caused by mural problems such as complete tracheal rings. TM may be localized or generalized. The mainstem bronchi may also be affected, which is referred to as tracheobronchomalacia (TBM). Less commonly, the mainstem bronchi and/or their distal divisions at the lobar or segmental level are affected alone, which is known as bronchomalacia (BM) (1–5).

The causes of airway malacia can be broadly divided into those congenital conditions that are associated with an excessively compliant or collapsible airway and those where the airway cartilage is found to be normal but is malformed due to a secondary or acquired cause. Tracheomalacia is the most common congenital tracheal abnormality with a reported incidence of 1 in 2,100 children (6), which is likely an underestimation given a wide spectrum of non-specific symptoms that are initially misdiagnosed (7). Primary or congenital TM/TBM can be found alone or in conjunction with other genetic and congenital disorders (5, 8, 9). Given the common origin of the trachea and esophagus during embryologic development, TM/TBM is a common respiratory problem in children who have esophageal atresia with or without tracheal-esophageal fistula (EA/TEF) (10–14). TM/TBM may also be associated with other airway and lung pathologies, such as laryngomalacia, laryngeal clefts, bronchopulmonary dysplasia, or cystic fibrosis (7, 15, 16). The airway malacia can be acquired from cartilage malformation caused by external compression from vascular abnormalities, such as vascular ring, pulmonary sling, or aberrant subclavian artery, or other mediastinal masses (17–20). Prolonged intubation, chronic infections, or inflammatory conditions may also cause TM/TBM (21–23).

## Anatomy

**Figure 1:**
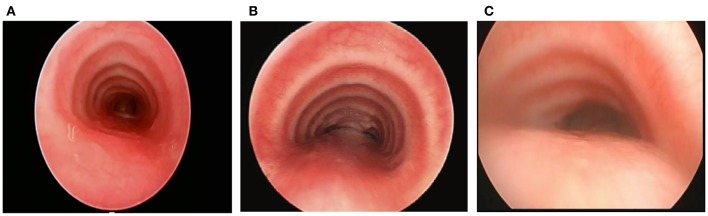
**(A)** Tracheal structure with normal C-shape rings. **(B)** U-shaped rings with a wider posterior membrane, demonstrating posterior intrusion. **(C)** Bow-shaped rings with a broad posterior membrane and severe posterior intrusion.

The normal trachea and main bronchi are supported by relatively rigid C-shaped cartilages anteriorly and laterally, and a short pliable posterior membrane (5). Changes in pressure, in part, determine the airway diameter during the respiratory cycle. The pliable posterior membrane moves inward during expiration, narrowing the airway and accelerating the airflow and mucus clearance. In a healthy person, the decrease in airway diameter is negligible and typically not more than 10–20% even with coughing, although some adult studies have shown up to 50% posterior intrusion with coughing by airway imaging and CT scans (24, 25). In patients with TM/TBM, the physiological narrowing of the airway is accentuated during expiration, and in severe cases, a clinically obvious airway collapse occurs predominantly when the expiratory effort is increased, such as during coughing or crying. The airway collapse may be attributable to the dynamic posterior intrusion and/or combined with a region of fixed anterior collapse. If the entire cartilage ring configures in an upside-down U shape or even bow shape, the posterior membrane is broader and more dynamic and intrudes into the airway lumen during expiration and periods of increased intra-thoracic pressure (Figure 1) (14). Also, intrinsic weakness of cartilages can have a profound effect on airway compliance; in the worst cases resulting in severe airway collapse at rest or with minimal exhalation effort. Anterior compression is typically caused by blood vessels such as the aorta or innominate artery, which may alter the shape of the cartilages even if they have normal strength leading to fixed anterior airway collapse.

## Symptoms and Signs

Clinical presentation of TM/TBM includes a range of non-specific respiratory symptoms, depending on the location, extent, and severity of the airway collapse. Many children with TM/TBM do not show symptoms until age 2–3 months (5, 26, 27). However, in cases with long-segment TM/TBM, symptoms may begin at birth (27). In some patients, TM/TBM becomes apparent soon after repair of EA/TEF when they cannot be extubated (27). A barking cough with expiratory rhonchi or inspiratory stridor may be present in most patients with TM/TBM. The extensive airway collapse can lead to ineffective cough and reduced clearance of secretions. As a result, patients with TM/TBM are at increased risk of frequent upper respiratory infections, prolonged recovery from an upper respiratory infection, and recurrent or persistent pneumonia (24, 28–31). Also, the airway obstruction often results in insufficient ventilation; therefore, patients may experience exercise intolerance, hypoxic episodes, or apneic events (5, 6, 27, 28). The symptoms can be worsened by any activities or conditions that increase the intrathoracic pressure and the patient's respiratory efforts, including activities such as coughing, crying, Valsalva maneuvers, feeding, forced expiration, or lying supine (5, 27).

## Diagnosis and Evaluation

There is no definitive standardized guideline for diagnosis and evaluation of TM/TBM. The diagnosis should be suspected by a clinical history of signs and symptoms that would be suggestive of TM/TBM, including barky cough, noisy breathing, recurrent pneumonia, prolonged pulmonary infection, feeding difficulties with dyspnea, cough, and aspiration, transient respiratory distress requiring positive pressure, oxygen dependence, ventilator dependence, blue spells, and apparent life-threatening events (ALTEs) (27, 28). Patients with apneic events require careful cardiac and neurologic evaluations to exclude these causes. Esophageal abnormalities, including strictures and tracheoesophageal fistulas, as well as gastroesophageal reflux, must be ruled out (27).

For the most accurate diagnosis of TM/TBM, direct visualization is achieved through flexible and rigid endoscopy, including laryngoscopy, tracheoscopy, and bronchoscopy (13, 24, 30, 31). Three-phase dynamic bronchoscopy is considered the gold standard for the diagnosis of TM/TBM. The first phase occurs while the patient is shallow breathing, which reveals the basic anatomy of the airway as well as compression, cartilage malformation, and secretion accumulation. It may also show vocal cord motion, depending on the depth of anesthesia. Vocal cord motion may be altered and even markedly depressed, sometimes differently on each vocal cord, with small amounts of anesthesia; this must be taken into consideration during the exam (13). The second phase is to induce coughing and Valsalva maneuvers while observing the entire airway, which reveals the maximum dynamic airway collapse as well as the secretion accumulation that gets displaced from the distal airways and comes into the larger airways. This phase is critical for identifying TM/TBM. The third phase is to distend the airways to 40–60 cm of water after aspirating all secretions out, which reveals the structures and lesions that may not normally be seen. This phase allows the identification of tracheoesophageal fistula (TEF), tracheal diverticulum, and aberrant bronchi (29) as well as regions of fixed compression. There are no standard criteria for establishing the diagnosis of TM/TBM endoscopically; however, most surgeons consider a more than 50% dynamic narrowing in the airway lumen with forced exhalation or coughing to diagnose TM/TBM (27). The majority of children with symptomatic TM/TBM have more than 75% airway collapse of one or more regions with forced exhalation or coughing, and those with recurrent pulmonary infections typically have complete collapse of one or more regions, causing impaired mucus clearance from the airway distal to that region.

**Figure 2:**
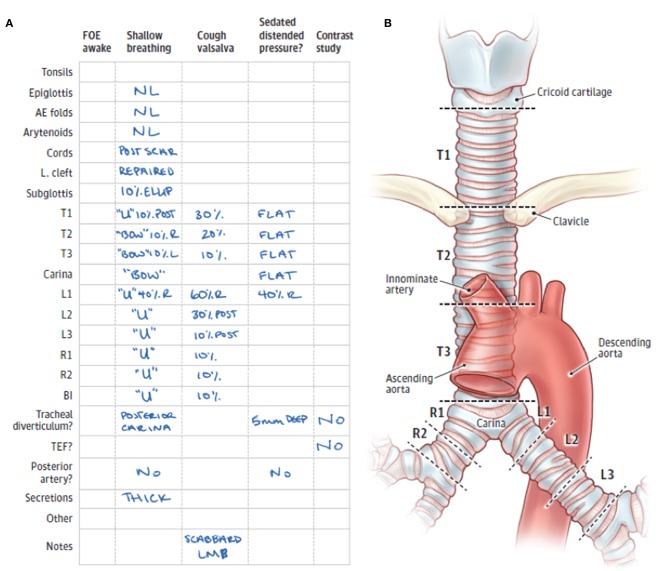
**(A)** Standardized form for documentation of 3-phase bronchoscopic findings. **(B)** Anatomic divisions of the trachea and mainstem bronchi to facilitate classification and description of tracheobronchomalacia. FOE, fiberoptic examination; AE, aryepiglottic; L cleft, laryngeal cleft; T1, trachea level 1; T2, trachea level 2; T3, trachea level 3; L1, left mainstem bronchus level 1; L2, left mainstem bronchus level 2; L3, left mainstem bronchus level 3; R1, right mainstem bronchus level 1; R2, right mainstem bronchus level 2; BI, bronchus intermedius; TEF, tracheoesophageal fistula; R, right-sided; L, left-sided; LMB, left mainstem bronchus; NL, normal; ELLIP, elliptical cricoid; U, U-shaped rings; BOW, bow-shaped rings [Illustration was adapted from Choi et al. (14) Copyright 2019 by American Medical Association].

Our team routinely uses a standardized reporting system for bronchoscopic evaluation based on anatomic regions and the severity of airway collapse. The airway has different surrounding structures throughout its course in the mediastinum. Taking these differences into account is important for a complete assessment of the airway as well as better communication with other providers. Anatomic regions are classified into the upper (T1), middle (T2), and lower (T3) trachea; and right and left mainstem bronchi. T1 is the upper third of the trachea, located above the clavicles and up to the cricoid cartilage (extrathoracic trachea). T2 is the middle third of the trachea, located below the clavicles to the takeoff of the innominate artery, which can usually be easily visualized during bronchoscopy. T3 is the lower third of the trachea, including the carina and the takeoff of the two mainstem bronchi. The right mainstem is divided into the proximal and distal right mainstem (R1, R2), and the left mainstem is divided into the proximal, middle, and distal left mainstem (L1, L2, L3). For each of these regions, we determine the percentage of airway narrowing and contribution of anterior collapse and/or posterior intrusion (13, 14) (Figure 2) as well as note any other airway distortion (such as lateral intrusion) or other airway lesions such as masses, cobblestoning, fistulas, etc.

**Figure 3:**
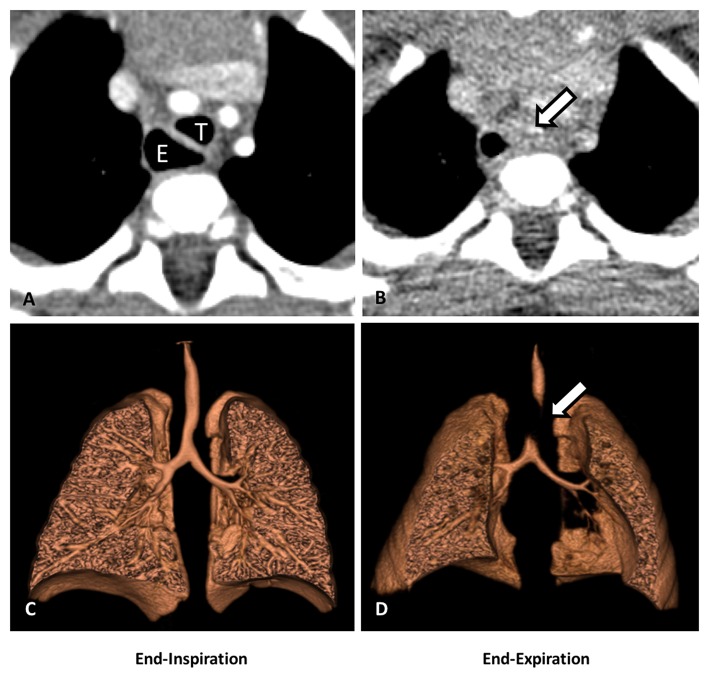
Dynamic airway CT scan with 3-D reconstruction. **(A)** CT scan (cross-sectional view): partial collapse of the mid trachea (T) and dilated esophageal dilation (E) at end-inspiration. **(B)** CT (cross-sectional view): complete collapse of the mid trachea (arrow) at end-expiration. **(C)** 3-D reconstruction of the airways: partial collapse of the mid trachea at end-inspiration. **(D)** 3-D reconstruction of the airways: complete collapse of the mid trachea at end-expiration (arrow).

In recent years, dynamic airway evaluation and angiography using a contrast-enhanced multidetector computed tomography (MDCT) with two-dimensional (2D) and three-dimensional (3D) reconstructions have become an important modality to aid in the evaluation of thoracic anomalies in pediatric patients (13, 14) (Figure 3). The dynamic airway evaluation can be performed in two phases of end-inhalation and end-exhalation (paired end-inspiratory and end-expiratory). Younger patients (usually under 6 years of age) may require sedation with laryngeal mask or intubation, but older patients can hold their breath (20, 32). Deep inhalation or breathe holding with 20 cm of water airway pressure is performed during the inspiration phase, and the exhalation phase is done with maximal exhalation or with zero airway pressure. The changes in the caliber of large airways are compared at both end-inspiration and end-expiration to identify the location of the TM/TBM caused by external compression (20, 33), but may not identify the severity of dynamic airway collapse due to the zero airway pressure used (as opposed to high end-expiratory pressures during coughing). Posterior membrane intrusion may create a “frown-sign” during forced exhalation (34). CT angiogram with 3-D reconstructions helps to evaluate vascular anomalies, if present, and the airway anatomic relationships to surrounding vasculatures. We also use a modified dynamic CT angiogram to identify the artery of Adamkiewicz to avoid injury to the spinal cord during surgery. MDCT study provides important information about the location and extent of TM/TBM, as well as the surrounding intra-thoracic structures and anomalies. This information helps surgeons to better understand the region of TM/TBM and the relevant surrounding structures, such as the location of the aorta and the innominate artery relative to the trachea, and the presence of any vascular anomaly that might alter the surgical plan. However, it is important to understand that a dynamic MDCT tends to markedly underestimate the degree of airway collapse (13, 27), and therefore, it cannot be used to “rule out” TM/TBM and needs to be employed in combination with diagnostic 3-phase bronchoscopy.

Other studies that are helpful in the preoperative assessment include an esophagram to assess swallowing, strictures, and aspiration, a ventilation-perfusion (V/Q) scan to assess lung performance, and an echocardiogram to confirm acceptable heart function prior to undergoing a large surgery (31). A newer study is the nuclear clearance study, which can assess the function of the esophagus, emptying of the stomach, aspiration, and tracheal clearance.

## Medical Management

Many physicians have the opinion that symptoms may improve within a few years without surgical intervention. All patients affected by mild to severe TM/TBM may benefit from medical management. The mainstay of medical management while awaiting airway structural stability is the optimization of the ciliary clearance of secretions since the cough clearance mechanism is thwarted by airway collapse (24, 28–31). In order to optimize airway clearance, ipratropium bromide (Atrovent) is administered to minimize the secretions without thickening the secretions as may occur with glycopyrronium bromide (Robinul). Normal saline or hypertonic saline is also nebulized to thin the secretions as much as possible. We have many patients with clinical signs of cough and recurrent respiratory infections who are effectively managed on a clearance regimen and can avoid surgery. Pulmonary hygiene (formerly referred to as pulmonary toilet) and chest physiotherapy to help with mucociliary clearance, as well as control of gastroesophageal reflux (GER) to minimize aspiration of inflammatory gastric contents, are also encouraged. In patients with a history of EA/TEF, the concern for esophageal dysmotility with stagnation and bacterial or fungal overgrowth and/or GER and the need for fundoplication must be seriously considered. Concerns for recurrent or congenital tracheoesophageal fistula leading to airway contamination should be investigated. In our opinion, routine and aggressive or continuous use of corticosteroids should be avoided due to the risks of cartilage degradation and progressive tracheomalacia. Continued exposure to steroids may cause tissue weakening and progressive small airway collapse. In addition, enthusiastic use of steroids can lead to Cushingoid appearance and adrenal suppression.

There is little evidence for the benefit of bronchodilators and muscarinic agents in patients with TM/TBM. While bronchodilators are widely used for wheezy patients with reactive airway disease, administrating a beta-agonist may worsen TM/TBM by reducing the tone of airway smooth muscle, resulting in a more pliable posterior membrane (35). On the contrary, muscarinic agonists such as bethanechol and methacholine can directly stimulate the airway smooth muscle and increase the posterior membranous tone (35). However, there is no evidence supporting the clinical efficacy of pharmacologic stimulation of airway muscle tone in patients with dynamic airway collapse caused by the posterior intrusion of the membranous component.

In the past, the initial approach in patients with severe TM/TBM was the placement of a tracheostomy and long-term mechanical ventilation. Tracheostomy is not a risk-free procedure, particularly in small infants, requiring changes in the size and length of the tracheostomy tube as the child grows. Even after successful tracheostomy placement, this approach may be associated with risk of tracheal injury, inflammation-causing granulation tissue, tracheo-arterial fistula formation, tracheal stenosis, tracheal pouch formation, tracheo-esophageal fistula formation, tracheal plugging, accidental decannulation, delayed vocalization, and difficulty with decannulation as the placement of the tracheal tube does not address the problem of the collapsible airway distal to the end of the tracheostomy tube. In addition, secondary TM and tracheal fibrosis from the presence of the tracheostomy may occur. In patients with TM/TBM that extends beyond the tip of the tracheostomy tube, the utility of the tracheostomy is to be questioned since the patient will still require positive airway pressures and may continue to have blue spells and recurrent infections that can cause progression of the TM/TBM.

## Surgical Treatment

A common misconception is that children outgrow TM/TBM. The truth is that TM/TBM is typically a congenital malformation of the tracheal cartilages and can sometimes slowly get less clinically serious over time, but TBM does not resolve on its own and can even get worse with age. Surgical treatment is reserved for the most severe cases and must be specific to the type and location of the TM/TBM in each patient based on the detailed diagnostic assessment protocol. All the associated conditions, such as cartilage deformation, vascular anomalies, mediastinal masses, tracheoesophageal fistula, abnormal airway branching as well as chest wall and spine deformity, should also be taken into account. Each operation needs to be customized to the airway anatomy, including decisions about the desired anatomic relationships of the trachea to the esophagus and major vasculature. Surgical options for the treatment of TM/TBM include pexy procedures (anterior aortopexy, anterior and/or posterior tracheopexy, anterior and/or posterior mainstem bronchopexy, posterior descending aortopexy), tracheal resection and end-to-end anastomosis or slide tracheoplasty, and placement of external splints and internal stents either absorbable or permanent (36).

**Figure 4:**
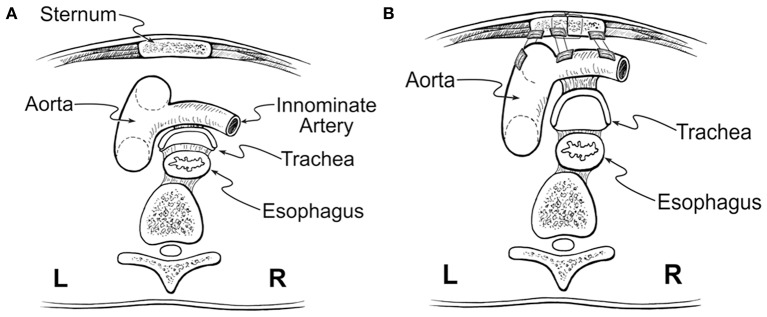
Anterior aortopexy. **(A)** Anterior collapse of the trachea, caused by compression of the aorta. **(B)** Anterior aortopexy with suspension of both the ascending aorta and the innominate artery to the posterior surface of the sternum using pledgeted horizontal mattress sutures. This image was illustrated by Dr. John Foker and published with his permission.

Tracheomalacia has historically been addressed with anterior aortopexy. This technique was derived from the operation described by Gross in 1948 to treat the innominate artery compression syndrome and was popularized by Filler et al. (37). Through the anterior approach, after removing the thymus, the ascending aorta or aortic arch and innominate artery pulled anteriorly and then sutured to the posterior surface of the sternum (30, 38) (Figure 4). The airway is loosely attached anteriorly to the major vessels by areolar tissue. Aortopexy relieves the anterior compression and supports the anterior wall of the trachea through tension on the areolar tissue. This technique can also be used to suspend the pulmonary artery, innominate artery, or pericardium if the anterior airway collapse persists (31). However, aortopexy does not directly address the airway pathologies associated with TM/TBM. Anterior aortopexy may not be a sufficient strategy in the patients with dynamic airway collapse caused by the posterior intrusion of the membranous component, which is the major contributor to airway collapse in many pediatric cases. In a recent meta-analysis, aortopexy was effective in clinically improving more than 80% of children; however, 8% showed no improvement, 4% showed worsening of their symptoms, and 6% died (30).

**Figure 5:**
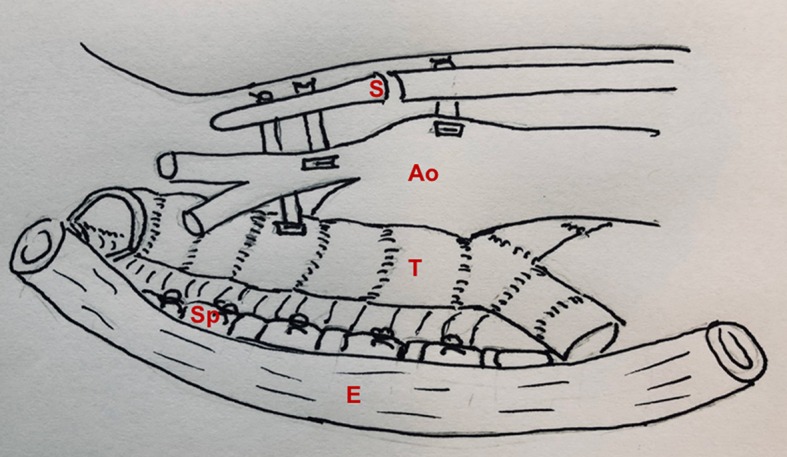
Posterior and anterior tracheopexy combined with anterior aortopexy. S, sternum; Ao, aorta; T, trachea; Sp, spine; E, esophagus.

To address this problem, our group developed anterior and posterior tracheobronchopexy to directly address anterior airway collapse and posterior membranous intrusion, respectively (28, 31, 39). Anterior tracheobronchopexy supports the anterior wall of the trachea and/or main bronchi to the sternum and anterior chest wall, whereas the posterior tracheobronchopexy fixes the posterior membrane to the anterior longitudinal spinal ligament (Figure 5). Tracheobronchopexy is done under direct bronchoscopic guidance to confirm the precision of suture placement by providing tracheal luminal visualization during suture placement and to avoid full-thickness sutures. To achieve optimal airway patency, patients may require airway procedures from both posterior and anterior approaches. The posterior work is preferred to be done first, allowing adequate scarring of the posterior tracheal membrane before pulling the anterior trachea in the opposite direction with anterior aortopexy and anterior tracheopexy. Our group has recently reviewed our first 98 patients who underwent posterior tracheobronchopexy through an open approach, proving bronchoscopic and clinical evidence of improvement in airway collapse (29). More recently, we have performed posterior tracheobronchopexy through thoracoscopic and robotic approaches for the treatment of TM/TBM in select individuals (40).

**Figure 6:**
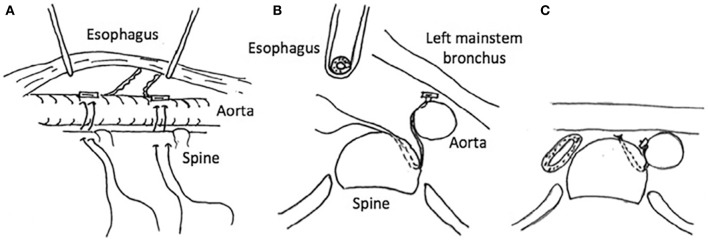
Posterior descending aortopexy. **(A)** Anatomic relationships of the descending aorta to the esophagus and the spine. **(B)** Cross-sectional view: the esophagus is rotated to the right and the descending aorta is moved to the left and secured to the side of the spine as posteriorly as necessary to relieve posterior pressure off the left mainstem bronchus. **(C)** Descending aortopexy sutures are tied, relieving left mainstem posterior intrusion and compression between the descending aorta and the pulmonary artery.

The effectiveness of posterior tracheobronchopexy can be limited if compression of the mainstem bronchus from the descending aorta is noted. The posterior descending aortopexy can be used to relieve left mainstem posterior intrusion and compression between the descending aorta and the pulmonary artery (41) (Figure 6). The posterior descending aortopexy can be performed from either the right or the left side. Most commonly, it is performed from the right side in patients with a left aortic arch as other airway work can be performed through the same incision. After mobilizing of the descending aorta, the posterior descending aortopexy is performed by passing autologous pericardial (or other tissue such as pleural or scar tissue) pledgeted polypropylene sutures to secure the aorta to the side of the spine, and as posteriorly as necessary to relieve posterior pressure off the left mainstem bronchus (41). This posterior movement of the aorta may necessitate dividing one or more intercostal arteries, and preoperative MDCT helps to avoid risking injury to the artery of Adamkiewicz.

The pediatric trachea tolerates less anastomotic tension but is more mobile than the adult trachea. Tracheal resection with either end-to-end anastomosis or as a slide tracheoplasty may be indicated in patients with some types of short-segment TBM, such as the congenital absence of cartilage or severe cartilage deformation. However, this approach is not widely used because TBM rarely affects a short segment of the trachea or bronchus.

Internal airway stents have been attempted to treat severe tracheobronchial stenosis or tracheobronchomalacia; however, the use of this technique in children has been limited due to serious complications including migration or fracture, erosion into nearby structures, formation of granulation tissue, difficult removal, and the need for additional dilations or stents, especially with patient growth (42–45). Therefore, this approach has fallen out of the favor but is still considered in highly selected patients with life-threatening airway obstruction who have failed other therapeutical strategies or in whom the risk-benefit analysis points to internal stent placement.

External splinting may offer airway support as an alternative to internal stenting in selected patients with life-threatening TM/TBM. External splints with autologous materials and prosthetic materials have been used to stabilize the malacic or deformed airway. Implantation of external prosthetic splint has raised concerns in terms of long-term effects and complications, including infection and erosion into nearby structures (46). An external splint made from the molded resorbable plate can be sutured around the airway, providing temporary airway support with full resorption predicted to occur within 1–3 years (47). This hopefully allows enough time for the cartilages to reform in a more favorable shape and allows for the growth of the airway in the pediatric patient. Interesting work in this field has been performed by Dr. Green's group in Michigan. They introduced both resorbable and permanent custom-printed external splints for the treatment of severe tracheobronchomalacia (47–50). Using a CT scan and custom software with 3-D reconstruction of the airways, a polycaprolactone or nylon splint is created and then secured around the trachea or bronchus to keep the airway open. A recent study published by this group reported the clinical efficacy of the 3D-printed bioresorbable airway splint device in a series of critically ill children with severe tracheobronchomalacia (50). The Esophageal and Airway Treatment (EAT) Center has been using the moldable bioresorbable plates (RapidSorb, Synthes CMF) to make intra-operative customized external splints in patients found to have airway compression or deformation not alleviated by anterior or posterior airway pexy placement. Many of these patients had complex airways which also required slide tracheoplasty or bronchoplasty, and/or anterior and posterior tracheobronchopexy.

## Conclusions

Tracheobronchomalacia is a clinically challenging condition, frequently undiagnosed or misdiagnosed in pediatrics, and includes many airway pathologies that cause either fixed or dynamic airway narrowing. Patients are best assessed and managed by a multidisciplinary team in centers specializing in complex pediatric airway disorders. The treatment plan should be individualized with a thorough approach to the underlying pathology and clinical concerns. All patients warrant aggressive medical management. For those children considered candidates for surgical intervention, three-phase dynamic bronchoscopy, and dynamic airway CT showing the position of vessels and other important landmarks in the mediastinum are helpful in surgical planning.

## Author Contributions

AK and RJ: study conception and design and critical revision. AK: drafting of the manuscript.

### Conflict of Interest

The authors declare that the research was conducted in the absence of any commercial or financial relationships that could be construed as a potential conflict of interest.
